# Algorithm-Guided Treatment of Ulna Impaction Syndrome: A 10-Year Follow-Up Study of Ulna Shortening Osteotomy and Wafer Procedure

**DOI:** 10.3390/jcm13133972

**Published:** 2024-07-07

**Authors:** Irene Mesas Aranda, Elisabeth Maria Haas-Lützenberger, Sara Imam, Riccardo E. Giunta, Elias Volkmer

**Affiliations:** 1 Division of Hand, Plastic and Aesthetic Surgery, University Hospital, LMU Munich, 80336 Munich, Germany; 2 Division of Vascular Surgery, Helios Klinikum Munich West, 81241 Munich, Germany; imam.sara@gmail.com; 3 Department of Hand Surgery, Helios Klinikum Munich West, 81241 Munich, Germany

**Keywords:** ulnar impaction syndrome, ulnar shortening osteotomy, wafer procedure, ulnar-sided wrist pain, ulnar variance, hand surgery

## Abstract

**Background**: Ulnar impaction syndrome (UIS) is a common degenerative wrist condition which results from positive ulnar variance, leading to an overload on the ulnar carpus. Ulnar shortening osteotomy (USO) and the arthroscopic wafer procedure (AWP) are established therapies for UIS if conservative management fails. This study assessed an algorithm-guided treatment of UIS over a period of 10 years. **Methods:** This prospective observational study compared the outcome of 54 patients who underwent either USO or AWP for UIS based on a predefined treatment algorithm. The mean follow-up period was 10 years. Primary outcome parameters were the visual analogue scale (VAS) for pain and the Disabilities of the Arm, Shoulder, and Hand questionnaire (DASH), whereas secondary outcome parameters were grip and pinch strength and range of motion. **Results:** The median preoperative ulnar variance was 2.6 mm in the USO group and 2.0 mm in the AWP group. The postoperative average ulnar variance was 0 mm in both groups. The preoperative pain at rest was 3.4 in the USO group and 2.3 in the AWP group. One year after surgery, there was a significant reduction to VAS 0.7 and 0.2, respectively. These results persisted to the 10-year follow-up (VAS 0.9 and 0.2). The pain in motion also decreased significantly in the first year (from 6.8 and 6.7 to 2.2 and 2.1), as well as after 10 years (2.4 and 1.0). The preoperative DASH score averaged 31.3 in the USO group and 35.8 in the AWP group. At the 10-year follow-up, the DASH of both groups decreased significantly to 4.35 in the AWP group compared to 12.7 in the USO group. **Conclusions**: Our data show that, when using our algorithm, both USO and AWP, two common operative treatment options of UIS, reliably reduce pain and significantly reduce the DASH score over at least a period of ten years. The results after 10 years differ from short-term results in so far as after one year, the USO group showed to some degree similar outcome parameters compared to AWP, whereas at the 10-year follow-up, AWP reached slightly better primary outcome parameters. The algorithm presented, thus, produced excellent short- and long-term outcomes. Our findings and the applied algorithm can assist in decision-making and patient education.

## 1. Introduction

Ulnar impaction syndrome (UIS) is a degenerative condition of the wrist that manifests with chronic ulnar-sided wrist pain. In UIS, the ulna is longer relative to the radius and, thus, exerts increased pressure on the surrounding structures within the wrist. Consequently, the articular disc may impinge between the ulnar head and the carpal bones, causing discomfort, pain, and, subsequently, damage to the triangular fibrocartilage complex (TFCC), the hyaline cartilage of the ulnar head as well as to the carpal bones, mainly the lunate and the triquetrum.

Ulnar positive variance may be congenital (idiopathic or associated with Madelung deformity) or due to radial pathology (malunion after a distal radius fracture, premature closure of the distal radial epiphysis, radial head resection, or following an Essex Lopresti injury) [1,2]. UIS may, thus, manifest in literally everybody’s wrist, yet it is especially prevalent among certain occupational groups and athletes engaged in activities that involve specific repeated wrist movements such as pronation–supination and axial loading. The repetitive stress of the ulna against the TFCC and the ulnocarpal bones results in progressive degenerative TFCC tears, disruption of the lunotriquetral ligament, distal radioulnar joint (DRUJ) instability, and wrist osteoarthritis [1,2].

In 1986, Palmer and Werner investigated the biomechanics of the DRUJ, demonstrating that on a wrist with neutral ulnar variance, the radius bears 81.6% and the ulna 18.4% of the load to the carpus. Increasing the ulnar length by 2.5 cm raises the force load to 41.9% while shortening the ulna by 2.5 cm reduces the load to 4.3% [3]. Based on this biomechanical study, the treatment for UIS involves decompressing the ulnocarpal joint by shortening the ulna [1,2].

The treatment of UIS typically involves a combination of conservative measures and, in more severe cases, surgical interventions. Initial management comprises conservative therapy, including non-steroidal anti-inflammatory drugs (NSAIDs), avoidance of specific movements (forced pronation, ulnar deviation, or power-gripping), splint immobilization, and steroid injections in selected cases. If conservative treatment fails, surgical therapy to decompress the ulnocarpal joint is indicated. The two most common surgical methods include ulna shortening osteotomy (USO) and the arthroscopic wafer procedure (AWP) [4,5]. Under specific circumstances, radial lengthening osteotomy or an open wafer procedure may be warranted.

USO, first described by Milch in 1941 [6], has undergone multiple improvements in technique and hardware over the last 100 years. However, complications, including non-union, malunion, ulnar deformity, distal radioulnar joint (DRUJ) arthritis, wrist stiffness from prolonged immobilization, hardware irritation, and significant scar formation, were not uncommon [7].

Feldon proposed a less invasive method in 1992 to treat TFCC tears with mild positive ulnar variance [8]. The open or arthroscopic wafer procedure (AWP) involves resecting 2–4 mm of the distal head of the ulna while preserving the DRUJ, ulnar styloid, and attached tendons. This procedure yields satisfactory results and avoids complications associated with internal fixation, such as non- or malunion or hardware irritation, thereby minimizing the need for secondary operations [7,9]. However, potential risks, including portal site infection, wrist empyema, extensor tendon inflammation (especially ECU tendinitis), wrist ganglion development, nerve or cartilage injury, DRUJ arthritis, and ineffective treatment, should be carefully considered [10,11,12,13]. If the ulna plus is more than 2–4 mm, the wafer procedure is not recommended, as it may result in substantial loss of the ulnar head and, thereby, impair DRUJ function.

USO using a plate inflicts less harm to the ulnocarpal joint as the TFCC and the hyaline cartilage of the ulna remain untouched. Furthermore, also cases of more than 4 mm of ulnar overlength may be treated without destroying the ulnar head and the DRUJ. In recent years, several companies came up with significant improvements in their ulna shortening plates, resulting in increased reliability regarding the actual extent of shortening as well as in far fewer non-unions. These improvements induced some kind of a paradigm shift, as the previously unreliable method of USO has now become an extremely reliable procedure.

Several clinical studies have compared USO to the AWP for treating UIS. However, to our knowledge, no study has assessed the clinical outcomes of these procedures after a minimum follow-up period of 10 years. Consequently, the primary objective of this study is to compare the long-term results of both procedures as viable treatment options for UIS, utilizing a follow-up period of 10 years. Additionally, it is worth noting that there is currently no established surgical algorithm for addressing UIS. Hence, our secondary aim was to analyse and validate a pre-established treatment algorithm based on clinical experience.

## 2. Materials and Methods

**Figure 1:**
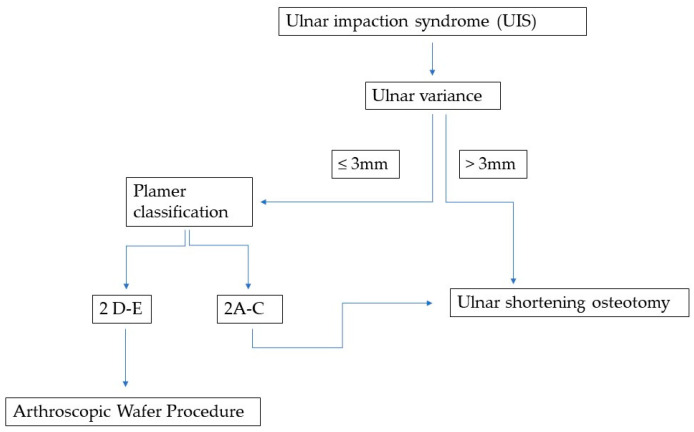
Treatment algorithm for ulnar impaction syndrome by Dr. Volkmer.

This prospective, monocentric, non-randomized, and non-blinded observational cohort study was conducted in our department for Hand and Plastic Surgery. We assessed 34 ulnar shortening osteotomies (USO) performed in 30 patients and 20 arthroscopic wafer procedures (AWP) carried out in 19 patients (Figure 1). All patients provided informed consent at the initiation of the study and during the final examination conducted 10 years after surgery. Ethical approval for the study was obtained from our institutional review board.

All patients underwent the same diagnostic procedures, which included a comprehensive clinical and radiological examination, along with wrist arthroscopy. Initial symptoms reported during the first visit (baseline) included ulnar-sided wrist pain, which was exacerbated during ulnar deviation and prono-supination of the wrist, power grip, and lifting off from a seated position. Local swelling was observed in most patients in both cohorts. The ulnar variance was assessed via X-ray imaging in posterior–anterior and lateral projection, with the arm positioned at 90° abduction [14]. MRI scans were utilized to identify TFCC injuries, carpal bone oedema, cysts, or ulnocarpal chondromalacia. Subsequently, wrist arthroscopy was performed to classify intraarticular damage, particularly TFCC injuries, using the Palmer classification [15].

Indications for surgery included a diagnosed UIS and failure of conservative treatments, which included splinting and NSAID therapy for at least 2–3 months.

All patients presenting to our clinic during the study period were enrolled if they met the following inclusion criteria:

- Diagnosis of ulnar impaction syndrome (UIS) based on clinical and radiographic evaluations.
- Agreement to surgical treatment after the ineffectiveness of conservative treatment options (splinting and NSAID therapy for at least 2–3 months).
- Patients aged 18 years and older.
- Willingness to participate and provide informed consent for the study.

The exclusion criteria were as follows:

- Medical contraindications to surgery or high risk for surgical complications.
- Pregnancy.
- Radial malunion more complex than just shortening of the radius.
- Other significant wrist pathologies were symptomatic at the time of evaluation.

All procedures were performed by a single experienced EBHS board-certified hand surgeon, the head attending of hand surgery in our department, which minimized variability in surgical technique. Patients were thoroughly informed about both conservative treatment options and the surgical treatment algorithm. The algorithm, developed from the long-term experience of the surgeon and supported by the existing literature, ensured a structured approach to determining the best treatment for each patient.

The algorithm-based grouping of patients into both surgical procedures was based on diagnostic findings. If ulnar variance was more than 3 mm, a USO was performed. If ulna variance was 3 mm or less, patients with a Palmer Type A-C lesion also received USO in an attempt to preserve the hyaline cartilage of the ulnar head, the (remaining) articular disc, and, thus, the integrity of the ulnocarpal and DRU joints. If the TFCC damage was Palmer D or E, and ulnar variance was maximally 3 mm, patients were assigned to the AWP group (Figure 1). Patients with radial malunions more complex than mere shortening were classified as radial malunion patients and, thus, not enrolled in this study.

When designing the algorithm, several cornerstones were considered to optimize patient outcomes. Although the AWP is less invasive, it can only safely resect 2–3 mm of bone without risking damage to the distal radioulnar joint (DRUJ). Thus, ulnar shortenings requiring a resection of 3 mm or more must be performed using an ulnar shortening osteotomy (USO). Additionally, the wafer procedure compromises the cartilage of the ulnar head and, if still intact, the articular disc. To preserve the integrity of the ulnocarpal joint, the algorithm avoids AWP in cases where the distal ulna cartilage and triangular fibrocartilage complex (TFCC) are intact. Consequently, for patients with an ulnar variance of 3 mm or less, the decision is based on the condition of the TFCC and ulnar head cartilage. If these structures are damaged, typically in elderly or posttraumatic cases, an AWP is performed. However, if the TFCC and ulnar head cartilage are intact, usually in younger patients, the algorithm favours USO to maintain the important stabilizing structures of the DRUJ.

All included patients signed the informed consent prior to surgery and were provided with all necessary details of the study, especially regarding the long term follow-up.

**Demographics**

**Figure 2:**
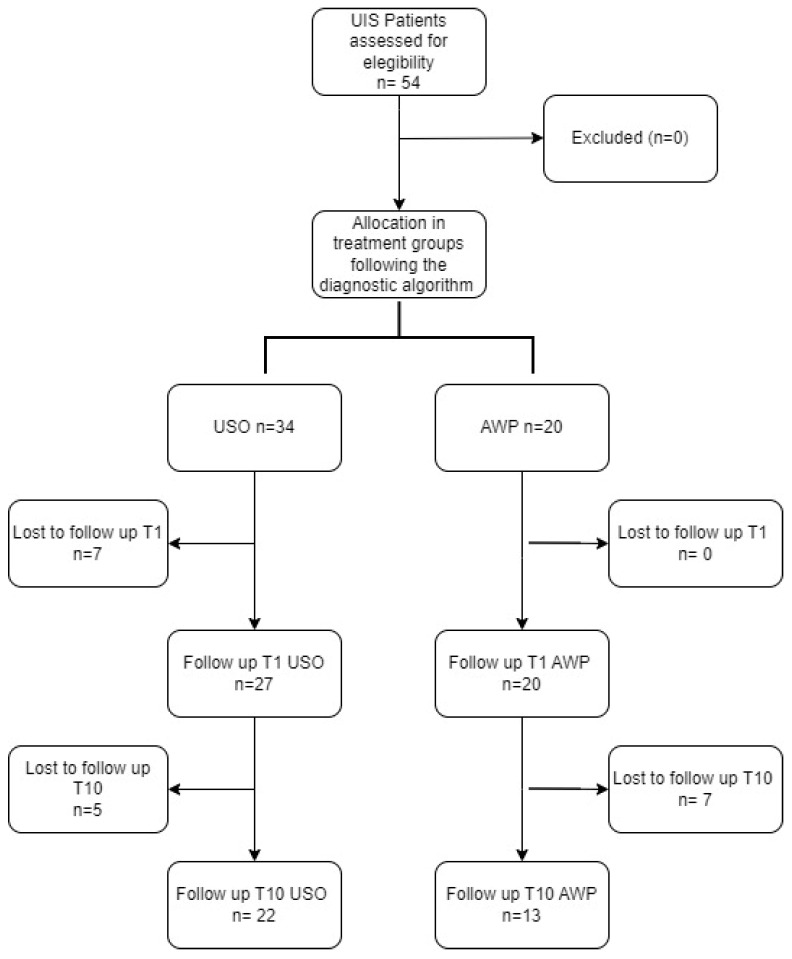
Study flow chart: Initially, 54 patients with ulnar-sided wrist pain and a positive ulnar variance in X-rays were screened for eligibility. Subsequently, 35 patients underwent the 10-year follow-up examination.

In the USO group, 34 patients were treated (29 females and 5 males), with a median age of 42 (36–57) years In the AWP group, a total of 20 patients were treated (10 females and 10 males), with a median age of 66 (42–79) (Figure 2). Detailed information about the baseline demographic characteristics is shown in Table 1.

Patients underwent clinical assessment prior to surgery (baseline), one year after surgery (T1), and at the 10-year follow-up visit (T10). These assessments included the visual analogue scale (VAS) to objectify pain at rest and during stress. Patient-reported functional outcome was analysed using the Disabilities of the Arm, Shoulder, and Hand (DASH) questionnaire. Additionally, grip strength (Jamar Dynamometer, Jamar Technologies, PA, USA) and pinch strength (Pinch Gauge Dynamometer PG-30, B + L Engineering, Santa Ana, CA, USA), as well as the range of motion (ROM) in both wrists were measured.

**Surgical technique**

Both the USO and the AWP were performed by the same experienced hand surgeon. Patients underwent either general or regional anaesthesia based on their medical history and preference. All patients received the diagnostic arthroscopy directly prior to the actual shortening surgery if not explicitly solicited otherwise by the patient.

**Wrist arthroscopy**

A complete dry diagnostic wrist arthroscopy was performed in all patients using a standard traction tower with 4 kg of traction. The 3–4 portal and the 6R portal were prepared. Diagnostic arthroscopy was executed with a 2.4 mm/30° wrist arthroscope, including assessment of all relevant ligaments and hyaline cartilage surfaces accessible, dorsal shaving of synovitis and floating remnants of the TFCC using a 2 mm shaver whenever needed. Finally, classification of the Palmer stage, including a precise assessment of lunate and triquetral bone chondromalacia, DRUJ and TFCC stability, TFCC wear, and other abnormalities, was performed.

**Ulnar shortening osteotomy (USO)**

For USO, a low-profile osteotomy plate specifically designed for ulna shortening osteotomy was used (Recos^®^, KLS Martin Group, Tuttlingen, Germany). A 7 cm ulno-palmar incision was made and preparation to the bone was carried out between the extensor carpi ulnaris and the flexor carpi ulnaris. While the muscle fascia and the M. pronator quadratus were incised where needed, the periosteum was preserved. The plate was positioned volarly, at least 3 cm proximally from the ulnar head and secured using the four preset drill sleeves. A 45-degree osteotomy was performed with an oscillating saw with permanent cooling after the saw guide had been fixated, and the exact amount of required shortening was defined. All ulnar shortenings were aimed at an ulnar neutral. After removal of the bone chip, closure of the osteotomy gap was achieved through compression of the plate and final fixation through insertion of two angular-stable screws. A lag screw was placed perpendicular to the osteotomy. Correct implant position and a satisfactory shortening were ensured by fluoroscopy. Postoperatively, the wrist was immobilized with a forearm splint for 4–6 weeks, depending on the expected compliance of the patient. Wrist exercises without weight bearing were allowed after 2 weeks; exercises with weight-bearing began after 6 weeks.

**Arthroscopic Wafer procedure (AWP)**

For AWP, diagnostic wrist arthroscopy was followed by a vigorous circular debridement of the TFCC. The resection of the ulnar head was performed with a burr while pronating and supinating the wrist in order to achieve an even resection surface. The insertion of the foveal TFCC was spared. The result was controlled via fluoroscopy. Postoperatively, a splint was administered to treat potential postoperative pain for a maximum of 14 days. Immediate mobilization and exercise of the wrist were allowed below the pain threshold. Weight-bearing was started 14 days after surgery.

**Statistical analysis**

Data are presented as means with standard deviation (SD) or medians with interquartile ranges (IQR) unless stated otherwise. Statistical analyses were performed using SPSS Statistics 28 (IBM, Armonk, NY, USA). Descriptive statistics were calculated for baseline (T0) characteristics and two follow-up visits (T1 and T10). All variables were tested for normality using the Shapiro–Wilk test. The tests were chosen based on the distribution of the variables.

Data were tested for distribution using the Shapiro–Wilk test. For within-group comparisons over time (T0, T1, and T10), the Friedman Test was used for non-parametric data. When significant differences were found, post hoc analysis was performed using the Wilcoxon Signed-Rank Test with Bonferroni correction for multiple comparisons. For between-group comparisons, the Mann–Whitney U Test was used for two independent groups, and the Kruskal–Wallis Test was employed for comparing more than two groups if the data were non-parametric. Categorical variables were analysed using Chi-square tests.

The level of significance was set at *p* < 0.05. When necessary, the Bonferroni correction was applied to adjust for multiple testing. Data were represented in tables or box plots, and all graphs were created using GraphPad Prism (Version 8.0.2; GraphPad Software, Inc., San Diego, CA, USA).

## 3. Results

### 3.1. Surgical Results

**Figure 3:**
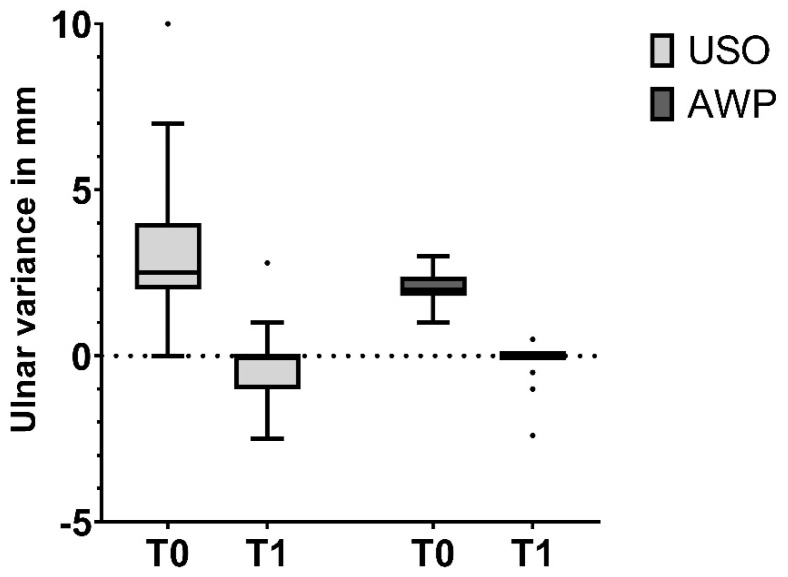
Mean ulnar variance before and after surgery.

**Figure 4:**
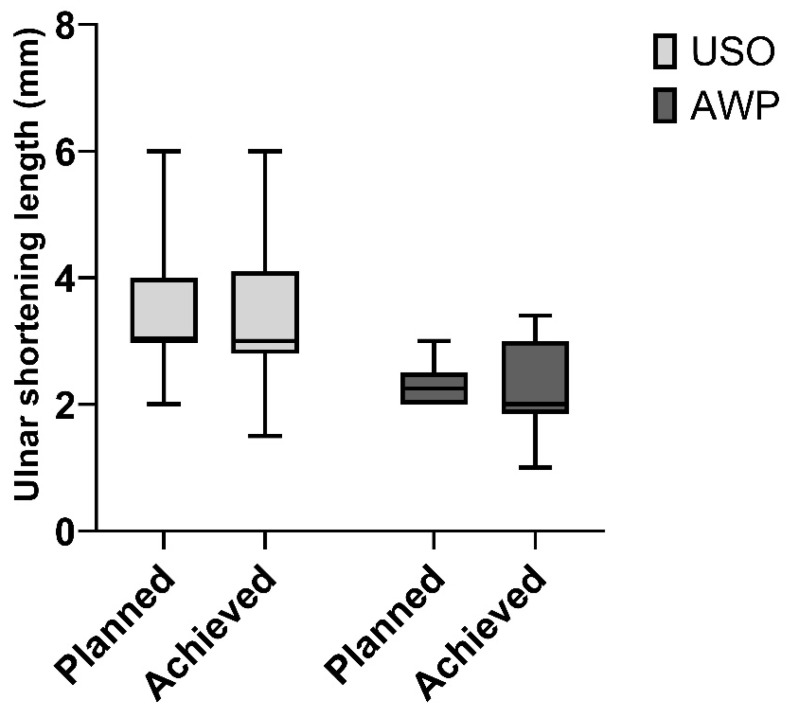
Planned and achieved ulnar shortening in mm.

The median preoperative ulnar variance in the USO group was 2.6 mm (IQR 2.0–4.0 mm), which decreased to a median of 0 mm (IQR −1.0–0.0 mm)) postoperatively (Figure 3). The targeted median shortening was 3.0 mm (IQR 2.7–4.6 mm), and the median achieved shortening was 3.0 mm (IQR 3.0–4.0 mm). In the AWP group, the preoperative median ulnar variance was 2.0 mm (IQR 1.8–2.4 mm), and it decreased to a median of 0 mm (IQR 0.0–0.0 mm) postoperatively. The median desired shortening was 2.3 mm (IQR 1.9–3.0 mm), and the mean achieved shortening was 2.0 mm (IQR 2.0–2.5 mm) (Figure 4). Comparing the operative time of both procedures, there was a significant difference. Specifically, USO took longer with a mean duration of 89 ± 32.6 min, contrasting with the AWP, which performed with an average duration of 64.8 ± 26.2 min (*p* = 0.007).

**Figure 5:**
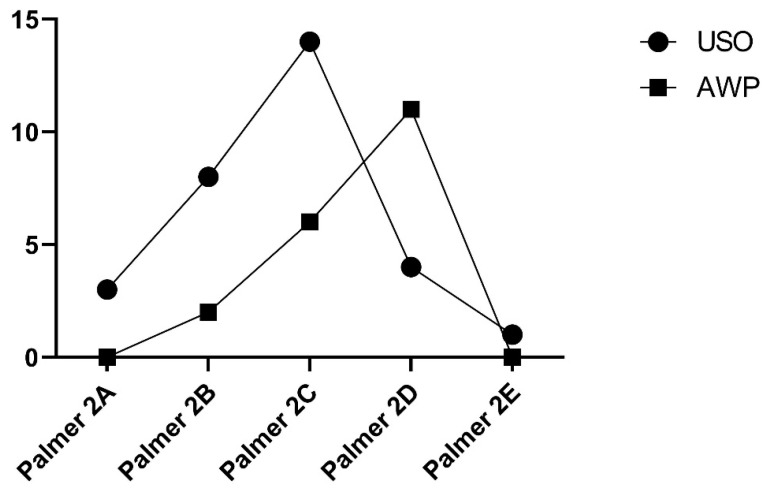
Palmer classification of TFCC lesion. In the USO cohort, predominantly Palmer 2C and 2B lesions were observed, while the AWP cohort exhibited a higher prevalence of Palmer 2D and 2C lesions during arthroscopy.

During the preoperative wrist arthroscopy of the USO group, 36% of the patients showed TFCC damage classified as Palmer 2C, 26% as Palmer 2B, and 10% as Palmer 2A. In the AWP cohort, 30% were diagnosed with Palmer 2C and 55% with Palmer 2D. Moreover, 40% of the USO cohort showed an intact TFCC (Figure 5).

There were no cases of non-union, delayed union, or malunion in the USO group. However, 62.5% of the patients in the USO cohort underwent hardware removal after approximately one year due to irritation. No complications occurred during or after metal removal.

### 3.2. Primary Outcome

The preoperative (T0) average VAS for pain at rest was 3.4 ± 2.9 in the USO group and 2.3 ± 2.5 in the AWP group. In motion, the preoperative pain measured was 6.8 ± 1.6 and 6.7 ± 1.8 in the USO and AWP groups, respectively.

**Figure 6:**
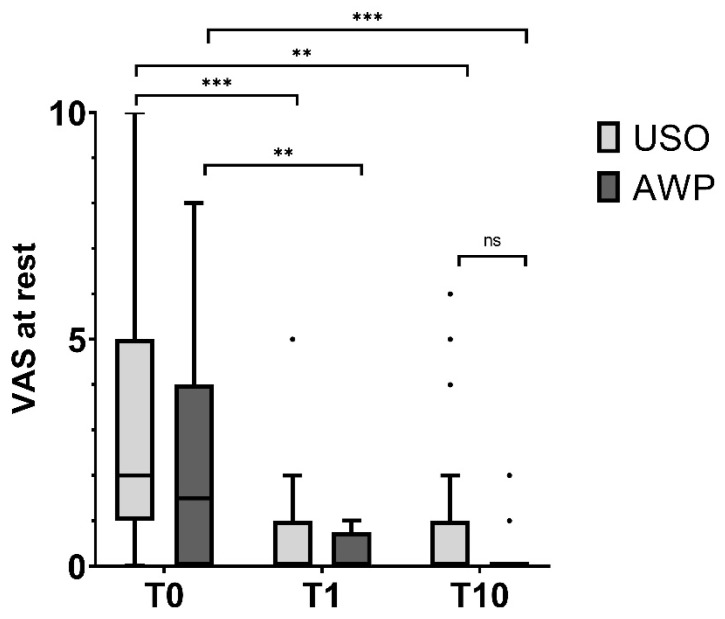
Pain at rest assessed by the Visual Analog Scale (VAS). There was a significant pain reduction in both cohorts at T1, which remained constant at T10. “ns” = not significant; ** *p* < 0.01; *** *p* < 0.001.

**Figure 7:**
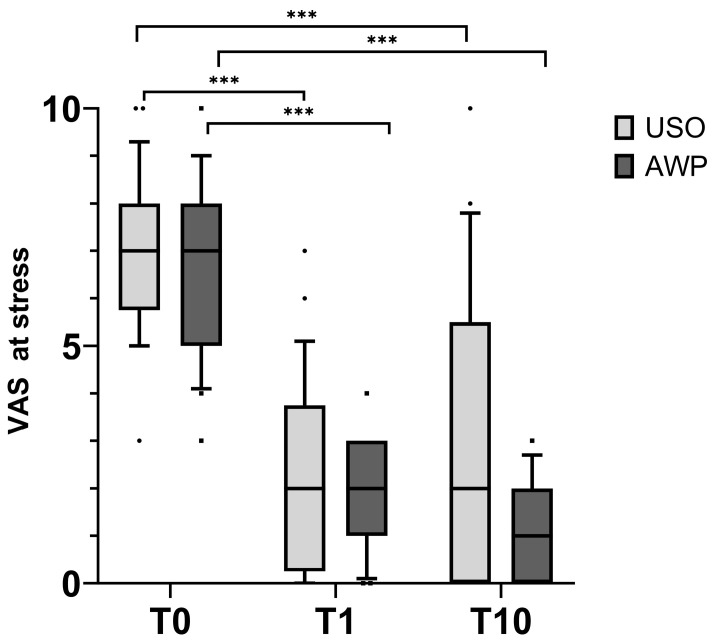
Pain under activity assessed by the Visual Analog Scale (VAS). Both cohorts experienced the same pain at T0, which was significantly reduced at T1. At T10, the AWP cohort experienced significantly less pain than the USO cohort. *** *p* < 0.001.

In the follow-up examination one year after surgery (T1), pain at rest significantly decreased to 0.7 ± 1.1 in the USO group (*p* = 0.003, Bonferroni adjusted) and to 0.2 ± 0.4 in the AWP group (*p* = 0.01, Bonferroni-adjusted) (Figure 6). In motion, the pain significantly decreased to 2.2 ± 1.9 in the USO group (*p* < 0.003, Bonferroni adjusted) and to 2.1 ± 1.1 in the AWP group (*p* < 0.003, Bonferroni-adjusted). In the long-term follow-up 10 years after surgery (T10), the pain at rest remained reduced compared to preoperative values in both groups (0.9 ± 1.7 in the USO group and 0.2 ± 0.5 in the AWP group), with no significant difference compared to VAS at T1 or between cohorts (Figure 7).

In the USO cohort, the VAS during activity was slightly higher (2.4 ± 2.7), yet with no significant difference compared to VAS at T1. However, in the AWP cohort, patients indicated further pain relief (1.0 ± 1.1) compared to the examination at T1 (*p* = 0.051, Bonferroni-adjusted) and also compared to the USO patients at the T10.

**Figure 8:**
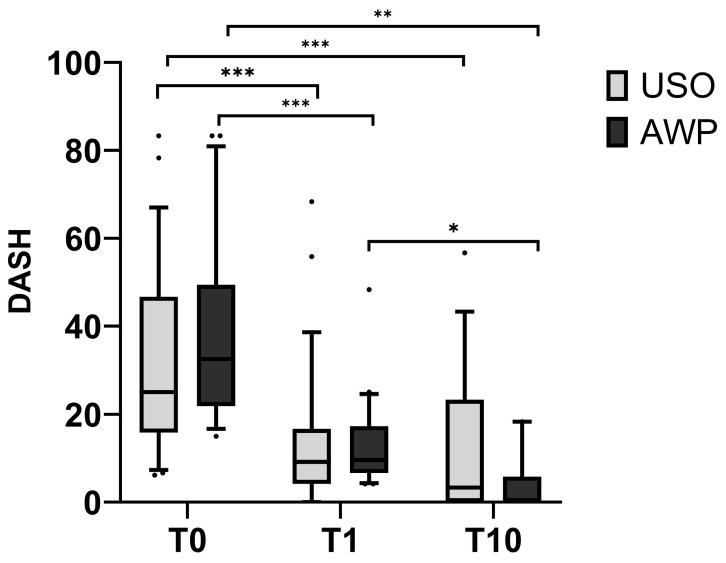
The DASH score at T0 was similar in both cohorts. At T1, both groups showed significantly lower DASH values, with a further decrease observed in the AWP cohort at T10. * *p* < 0.05; ** *p* < 0.01; *** *p* < 0.001.

According to the perceived pain levels, the patient-reported functional outcome, displayed by DASH scores at baseline (T0), were comparable in both cohorts (31.3 ± 20.6 for USO and 35.8 ± 19.5 for AWP). At T1, they decreased to 14.2 ± 16.2 in the USO group and 13.3 ± 9.8 in the AWP group, which was statistically significant (*p* < 0.003, adj.) in both groups. No statistical difference was observed between the groups. At the 10-year follow-up (T10), the average DASH score in the USO group remained similar at 12.7 ± 17.1; however, in the AWP group, it further decreased to 4.3 ± 6.3, with statistical significance (*p* = 0.15, adjusted for multiple testing) (Figure 8).

### 3.3. Secondary Outcomes

#### 3.3.1. Grip Strength

**Figure 9:**
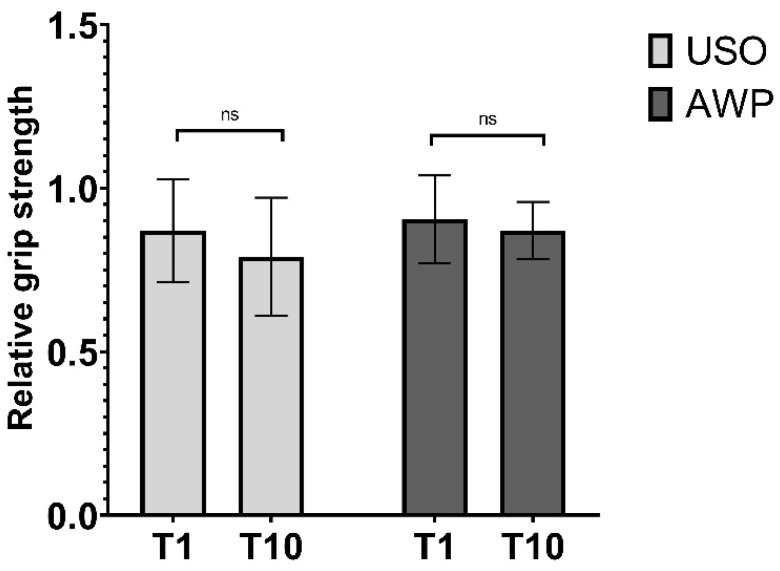
Relative grip strength presented as a percentage of the maximum grip strength measured in the contralateral hand. No difference was found between groups or time points. “ns” = not significant.

Maximal grip strength at T1 averaged 87.1% ± 15% of the contralateral hand in the USO group and 90.5 ± 13% in the AWP group. At T10, USO patients achieved 79% ± 18% of the measured maximal grip strength of the contralateral non-operated hand, and AWP patients achieved 87% ± 13% power grip strength of their contralateral hand. No significant differences between groups or time points were demonstrated (Figure 9).

#### 3.3.2. Pinch Strength

**Figure 10:**
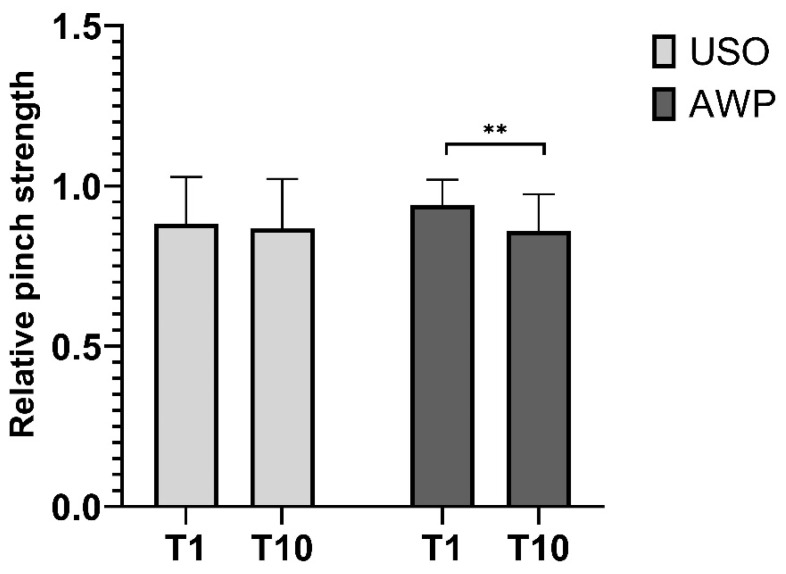
Relative pinch strength presented as a percentage of the maximum pinch strength measured in the contralateral hand. Patients in the AWP group had significantly less strength at T10 compared to T1. No difference was found between groups. ** *p* > 0.01.

Pinch strength at T1 was 88% ± 14% of the contralateral hand in the USO group, remaining consistent at T10 after 10 years (87% ± 15% of the opposite hand). AWP group patients achieved 94% ± 7% of the healthy hand at T1. At T10, a relative strength reduction to 86% ± 11% of the contralateral hand was observed. There was no significant difference in both groups (Figure 10).

#### 3.3.3. Wrist Mobility

**Figure 11:**
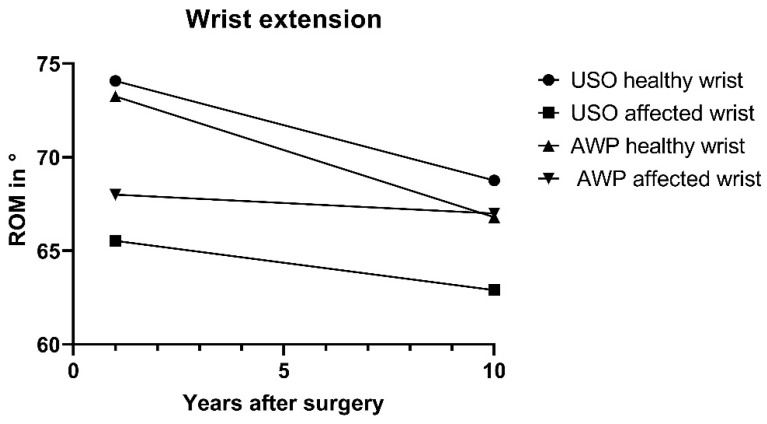
Wrist extension in °. USO Patients showed a greater extension deficit which stayed constant through the years.

Regarding the range of motion of the wrists, no statistically significant differences were observed between groups. At T1, 40% of patients in the USO group showed an average extension deficit of 20° ± 12° compared to the contralateral wrist. In the AWP group, 45% of patients had a mean extension deficit of 10° ± 6° at T1. In the 10-year follow-up (T10), 35% of the patients in the USO group had an extension deficit of 12 ± 9°, while in contrast, an extension deficit of 10 ± 7° was measured in only 15% of patients in the AWP group (Figure 11).

At T1, 40% of patients in the USO cohort had a flexion deficit of 21° compared to the opposite side. Ten years later, 35% had a mean flexion deficit of 14 ± 7° compared to the opposite side. In contrast, 60% of patients in the AWP group had a flexion deficit of 9 ± 6°, which was present in only 25% of patients at T10.

A total of 25% of the USO group had an ulnar adduction deficit of 13 ± 5° at T1, which remained similar 10 years later. In the AWP group, 20% of patients had an ulnar adduction deficit of 8 ± 2° at T1, and no difference could be detected at T10. Moreover, 15% of the USO patients had a radial adduction deficit of approximately 15° at T1. At T10, a deficit of 11° could be detected in 23% of the patients. In contrast, in the AWP group, a radial adduction deficit of 5° was detected in 30% of the patients at T1, and only 10% of the patients had a 5° radial abduction deficit at T10.

#### 3.3.4. Return to Work

**Figure 12:**
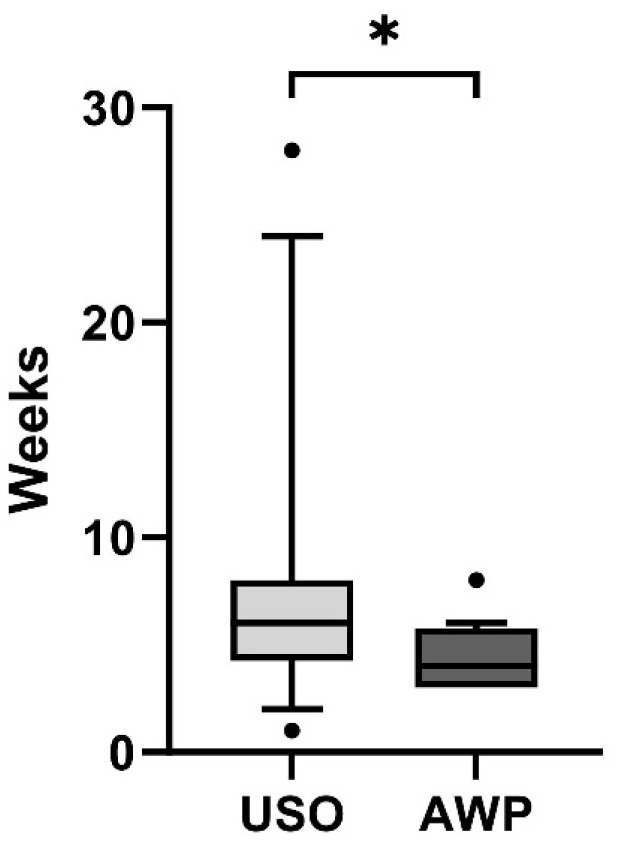
Postoperative work resumption in weeks. Patients in the USO cohort had a significantly longer work resumption period than AWP patients (* *p <* 0.01).

In terms of postoperative work resumption, individuals in the USO cohort required an average of 8 ± 6.7 weeks, displaying a notable variance. In contrast, patients undergoing the wafer procedure returned to work in an average of 4.3 ± 1.4 weeks (*p* = 0.01; Figure 12).

Within the USO cohort, a certain number of patients (n = 3) necessitated a change in their employment due to an inability to sustain their previous work, a situation that did not occur among any patients in the AWP group.

Primary factors contributing to this transition included persistent pain, diminished strength, and limitations in range of motion. Within the USO cohort, 54% held office positions, 27% engaged in strenuous manual labour, and 18% performed lighter manual tasks. In contrast, in the AWP cohort, 50% held office positions, 20% were in hard labour positions, and 30% performed lighter manual tasks (Supplementary Table S2).

### 3.4. Lost to Follow-Up Analysis

A total of 54 patients were included at baseline, with 35 completing the follow-up after 10 years, resulting in a follow-up rate of 65% and a drop-out rate of 35% (Figure 2). Among the 19 patients lost to follow-up, the reasons were moving residence to a different city (10%); inability to be contacted by telephone, e-mail, and mail; personal reasons resulting in a lack of time (23%); and advanced age (10%). All patients who were reached via telephone expressed gratitude and satisfaction with the procedure.

The demographic and baseline characteristics were similar between the follow-up and drop-out groups. The same proportion of the USO and AWP groups were lost to follow-up (35%). The mean age in the follow-up group was 53 years (median 55, IQR 39–66), while in the drop-out group, it was 52 years (median 46, IQR 42–62). Both groups had a similar gender distribution, with a predominance of females. Smoking status and the distribution of the affected hand (dominant or non-dominant) were also comparable. However, there was a higher proportion of patients with their dominant hand affected who were lost to follow-up.

Clinical outcomes indicated greater improvement in the follow-up group compared to the drop-out group. The mean VAS at rest decreased from 2.70 to 0.3 in the follow-up group and from 2.9 to 1 in the drop-out group. The mean VAS at stress decreased from 6.9 to 1.9 in the follow-up group and from 6.3 to 2.8 in the drop-out group. The DASH scores improved from a mean of 31 to 13 in the follow-up group and from 36 to 16 in the drop-out group. The relative grip strength at T1 was similar in both groups, with the follow-up group at 88% and the drop-out group at 89%.

A binary logistic regression model was performed to assess the influence of these variables on follow-up status. Most variables were not significant, but VAS at rest at T1 was significant. The Omnibus Test result was 0.008, with an odds ratio of 2.86 (95% CI 1.13–7.26) and a regression coefficient of 1.054 (*p* = 0.02), indicating that higher VAS at rest at T1 increased the likelihood of being lost to follow-up.

## 4. Discussion

Ulnar impaction syndrome (UIS) is a degenerative condition of the wrist caused by a relatively long ulna, which leads to an increased weight load and continuous ulnar-sided wrist impaction, causing progressive degenerative pathologies [1,2,3,16].

The gold standards for the treatment of UIS are the ulnar shortening osteotomy (USO) and the arthroscopic wafer procedure (AWP).

Numerous studies in the literature have undertaken a comparative analysis of both ulnar USO and AWP [17,18,19,20], reporting satisfactory outcomes for both surgical interventions. Nevertheless, studies describing and comparing the long-term results of both USO and AWP procedures are scarce. Addressing this gap in knowledge is crucial for our understanding of the optimal treatment strategy for UIS.

This long-term follow-up study provides evidence that both USO and AWP are highly effective treatment options for UIS. Informed by our experience and the existing literature [21,22], we employed a treatment algorithm that guided our choice between USO and AWP, considering insights from earlier diagnostic steps. If diagnostic arthroscopy revealed a Palmer lesion worse than 2C and an ulnar variance less than 3 mm, we performed an AWP in the same session. Patients with a Palmer type less than or equal to 2C and an ulnar variance greater than or equal to 3 mm underwent USO. This approach for treating UIS based on individual patient characteristics showed promising results, as both cohorts were almost pain-free at rest as well as at stress at T1, which remained low ten years later.

In our study population, nearly 70% of the patients had a perforated TFCC, which strongly correlated with UIS [23]. In the AWP group, 55% had a Palmer 2D lesion, 30% had a Palmer 2C lesion, and almost 40% of the USO group presented with an intact TFCC in the preoperative arthroscopy.

Regarding the planned USO, we aimed for 4 mm (3.68 ± 1.5 mm achieved) and 2.5 mm in the AWP group (2.1 ± 0.5 mm achieved). Only one patient in the AWP group required a USO due to insufficient shortening. Following this preset treatment algorithm, we did not experience any major complications, and most patients achieved satisfactory results.

Before surgery (T0), USO patients reported more pain at rest than AWP patients. Both groups experienced similar levels of pain during activity. This difference may be explained by the fact that USO patients had a higher ulnar variance, resulting in more pain at rest, while AWP patients showed higher TFCC damage (higher carpal damage in Palmer 2D- LT-Ligament perforation), which may have accounted for the similarity in pain levels during stress.

Regarding our primary endpoints, at T1, both cohorts were significantly pain-free at rest and reported very low levels of pain during stress (VAS 2–4). The reported pain levels at T10 remained constant in the USO cohort but showed a further decrease in the AWP cohort. A similar trend was observed in the DASH score. This difference may be attributed to the fact that USO patients experienced hardware irritation and a more prolonged healing phase after surgery, involving longer immobilization and increased demand for physiotherapy. These factors are more likely to introduce a psychological component influencing the subjective results of the UIS therapy. It also has to be kept in mind that patients receiving an AWP never had an ulnar positive variance higher than 2 mm, and the AWP was rarely performed when the TFCC was not yet perforated. This resulted in a selection of elderly patients with less ulnar length in the AWP group, which may also have accounted for the discrepancy in the long-term results. Overall, however, the difference between cohorts at T10 (VAS at stress 2.4 vs. 1.0 and DASH 12.7 vs. 4.3) is very small, emphasizing the promising results of both procedures.

In our study, females accounted for 72% (n = 39) of the total patients, which is a significant proportion. This distribution was particularly notable in the USO group, where 85% of the patients were female, compared to an equal gender distribution in the AWP group (50% female). This discrepancy necessitated a gender-adjusted analysis of the primary and secondary outcomes to ensure an accurate interpretation of the data Supplementary Table S1).

Upon conducting the gender-adjusted analysis, we observed that females in the USO group had a smaller preoperative ulnar variance (mean 3.0, SD 1.5) compared to males (mean 4.0, SD 3.4). However, in the AWP group, the ulnar variances were similar between genders. This difference in the USO group is likely biased due to the preallocation algorithm, which was based on ulnar variance preoperatively. This preallocation could have inadvertently resulted in more females being assigned to the USO group, influencing the observed outcomes. Despite ongoing refinements in surgical techniques and hardware [24], USO remains associated with a certain incidence of complications, especially delayed union, non-union, or hardware irritation. Some studies report non-union rates of up to 10% and a plate removal incidence of 70% [25]. Factors associated with delayed union are performing a transverse osteotomy instead of an oblique osteotomy and generating increased too much heat during the osteotomy [24,26,27,28]. With the described technique, we prevented these issues, especially by using the latest generation of ulnar osteotomy plate (Recos, KLS Martin). The Recos ulna shortening plate incorporates a locking compression plate, enabling precise and atraumatic osteotomy. Additionally, it features a low profile and round edges to minimize soft tissue irritation [27]. In our study population, which included 25% active smokers, we did not observe any case of non-union.

Despite these favourable outcomes, the majority of treated patients in our cohort opted for hardware removal due to irritation (62.5%). Patients reported pain, discomfort, limitations in daily activities, and cold intolerance. Following hardware removal, patients experienced improvement; however, at the 10-year follow-up (T10), they exhibited an almost identical mean DASH score compared to those who retained the hardware (14.3 ± 19.6 vs. 14.5 ± 17.9). In our results, hardware removal showed no significant impact on results in VAS for pain under stress (2.3 ± 2.6 after removal and 2.6 ± −3.2 with plate in situ). It has to be mentioned that Germany has a longstanding tradition of plate removal, even when there is no proven medical benefit. This practice significantly influences patients’ opinions, as they frequently request plate removal within a year. This tradition likely influences both patients’ and surgeons’ decisions regarding whether or not to remove plates.

The AWP avoids complications like non-union, hardware irritation, or nerve injuries.

Yu et al. published a systematic review and meta-analysis comparing the short-term outcomes of USO and AWP. They demonstrated that patients exhibited better grip strength when treated with AWP [29]. In contrast, Bernstein et al. and Stockton et al. could not establish any difference in the measured relative strength, with both cohorts showing around 84–87% of the contralateral hand strength, aligning with our findings [4,16]. Patients treated with AWP in our study had slightly better relative grip strength compared to those who underwent USO, both at T1 and T2 (T1 AWP: 94 ± 7% (median 95% (88–100%) and USO 88 ± 14% (median 94% (86–100%) and T2 AWP: 86 ± 11% (median 100% (87–116%), USO 87 ± 15% (median 96% (85.104%)). The differences in relative grip strength were minimal and not statistically significant, likely due to a small sample size. Limitations regarding strength measurement include whether the injured hand was also the dominant hand and if patients had additional injuries in the measured as well as in the contralateral hand.

The follow-up analysis revealed no significant differences in demographic and baseline characteristics between the follow-up and drop-out groups, indicating that the groups were comparable. The reasons for loss to follow-up were primarily related to the extended follow-up period, including relocation to a different city, older age, lack of time or interest, decreased willingness to participate, and incorrect or outdated contact information. These factors suggest that the drop-outs were not due to negative outcomes or dissatisfaction with the treatment but rather logistical and personal circumstances associated with the long duration of the study. This reduces the potential for bias in our results, as the loss to follow-up does not appear to be related to the effectiveness or satisfaction with the intervention (Table 2).

Further research with larger study samples is needed. A randomized controlled study directly comparing USO and AWR would provide high-quality results with minimized selection bias, as our study only included within-group analysis. Future research should also examine the influence of patient-specific characteristics, return to work, and sex in more detail. Additionally, it would be valuable to investigate X-rays after longer follow-up periods to assess for DRUJ arthritis, as the algorithm presented does not differentiate between the different shapes off the DRUJ.

Although our study shows slightly better long-term results in the AWP cohort, it is important to mention certain limitations of the AWP, such as the maximum achievable shortening length and the necessity for a damaged TFCC to access the ulna head. Consequently, using AWP to address a positive ulnar variance with an almost intact TFCC (e.g., Palmer 2A-B) could be a matter of debate.

## 5. Conclusions

Our long-term results confirm that the algorithm introduced by the attending physician of our department, Dr. Volkmer, reliably selects an effective individual treatment, as all patients showed improvement based on within-group analysis. The algorithm-assigned surgical intervention led to a definite pain reduction over 10 years, independent of the type of procedure performed.

## Figures and Tables

**Table 1:** Demographic characteristics of participants at baseline.

Baseline Characteristic	USO	AWP
	N = 34	N = 20
Gender		
Female (n (%))	29 (85)	10 (50)
Male (n (%))	5 (15)	10 (50)
Age (Median (IQR))	42 (36–57)	66 (42–79)
Affected Hand		
Right (n (%))	13 (38)	8 (40)
Left (n (%))	17 (50)	11 (55)
Both (n (%))	4 (12)	1 (5)
Dominant hand (n (%))	13 (40)	11 (52)
Aetiology		
Idiopathic (n (%))	21 (62)	15 (75)
Madelung Deformity (n (%))	3 (9)	
Distal radius fracture (n (%))	10 (29)	5 (25)
Smoking status		
Smoker (n (%))	11 (32)	3 (15)
Non-Smoker (n (%))	23 (68)	17 (85)
Ulnar variance (mm)		
Preoperative (Median (IQR))	2.6 (2.0–4.0)	2.0 (1.8–2.4)
Postoperative (Median (IQR))	0 (−1.0–0.0)	0 (0.0–0.0)
Pain (VAS)		
At rest (Median (IQR))	2.0 (1.0–5.0)	1.5 (0.0–4.0)
During stress (Median (IQR))	7.0 (5.6–8.0)	7.0 (5.0–8.0)
DASH-Score (Median (IQR))	25 (16–47)	32 (22–50)

Abbreviations: USO: ulnar shortening osteotomy; AWP: arthroscopic wafer procedure; IQR: interquartile range.

**Table 2:** Demographic characteristics of participants in the loss to follow-up analysis.

		Follow-Up					Drop-Out		
Variable		AF ^1^	RF ^2^	Mean (SD)	Median (IQR)	AF ^1^	RF ^2^	Mean (SD)	Median (IQR)
Procedure	USO	22	65%			12	35%		
	AWP	13	65%			7	35%		
Age				53 (17)	55 (39–66)			52 (17)	46 (42–62)
Gender	Female	25	66%			13	34%		
	Male	10	67%			5	33%		
Smoking status	Smoker	9	60%			6	40%		
	Non-smoker	26	68%			12	32%		
Affected hand	dominant	15	58%			9	42%		
	non-dominant	20	69%			9	31%		
VAS at rest	VAS T0			2.7 (2.6)	2 (0–5)			2.9 (3)	2 (1–5)
	VAS T1			0.3 (0.5)	0 (0–0.8)			1 (1.4)	1 (1–2)
VAS at stress	VAS T0			6.9 (1.5)	7 (6–8)			6.3 (1)	6 (5–8)
	VAS T1			1.9(1.5)				2.8 (1.9)	3 (1–4)
DASH	DASH T0			31 (17)	25 (20–42)			36 (25)	27 (18–51)
	DASH T1			13 (13)	9 (6–17)			16 (17)	12 (6–18)
Relative grip strength at T1 (healthy hand)				88% (13%)	90% (82–100%)			89% (15%)	93% (86–99%)

^1^ Absolute frequency (AF); ^2^ relative frequency (RF) of the follow-up/drop-out portion of the variable; standard deviation (SD); interquartile Range (IQR).

## Data Availability

The data presented in this study are available on request from the corresponding author due to ethical and legal reasons.

## Supplementary Materials

The following supporting information can be downloaded at: https://www.mdpi.com/article/10.3390/jcm13133972/s1, Table S1: Primary and secondary outcome- gender adjusted. Table S2. Information on patients occupations.
