# Supplementary material for: Algorithm-Guided Treatment of Ulna Impaction Syndrome: A 10-Year Follow-Up Study of Ulna Shortening Osteotomy and Wafer Procedure
Source: J Clin Med. 2024 Jul 7;13(13):3972. doi: 10.3390/jcm13133972 (PMC11242476; doi:10.3390/jcm13133972)
Supplement: Supplementary file 1 [file jcm-13-03972-s001.zip › jcm-3040873-supplementary.pdf]

Supplementary material

Table S1- Primary and secondary outcome- gender adjusted

Variable	Male				Female			
	USO		AWP		USO		AWP	
	Median	IQR	*Median*	IQR	*Median*	IQR	*Median*	IQR
Preoperative ulnar variance (mm)	3.0	2.0-6.5	2.0	1.7-2.2	2.5	2.0-4.0	2.0	1.7-3.0
Ulnar shortening (mm)	3.0	2.0-6.3	2.3	2.0-3.0	3.0	2.5-4.7	2.0	1.7-2.6
Pain at rest								
VAS T0	2.0	0.5-2.0	0.5	0.0-2.5	2.0	1.0-5.0	2.5	0.8-5.8
VAS T1	0.0	0.0-0.5	0.0	0.0-0.3	0.0	0.0-1.5	0.0	0.0-1.0
VAS T10	1.0	0.0-1.0	0.0	0.0-0.5	0.0	0.0-1.3	0.0	0.0-0.0
Pain during sdtress								
VAS T0	5.0	2.0-6.5	5.5	5.0-7.3	7.0	6.0-8.0	7.5	6.8-9.0
VAS T1	2.0	1.0-3.52	2.0	1.0-3.0	2.0	0.0-4.0	3.0	1.5-3.0
VAS T10	2.0	0.0-2.0	1.0	0.0-2.25	1.5	0.0-6.3	0.0	0.0-2.0
DASH Score								
T0	22	10-25	24	21-32	27	16-51	43	32-65
T1	9	3-37	9	7-14	9	4-17	14	6-18
T10	3.33	3.3-3-3	0	0-4.6	7	0-34	4	0-18
Relative grip strenght								
T1	92%	68-96%	93%	90-100%	95%	86-100%	90%	81-95%
T10	94%	75-94%	95%	77-108%	86%	74-103%	88%	80-99%
Relative pinch strenght								
T1	93%	90-98%	98%	93-100%	94%	80-100%	92%	86-100%
T10	85%	79-85%	96%	68-120%	96%	87-106%	100&	92-117%

Table S2. Information on patients occupations

The recorded occupations of patients include a variety of roles with differing physical demands. Here is a breakdown of the occupations by percentage of the recorded patients (24/54):

- Office Worker: 15% (n=4)
- Nurse: 19% (n=5)
- Gardener: 8% (n=2)
- Physiotherapist: 4% (n=1)
- Social Service Worker: 4% (n=1)

• Salesperson: 4% (n=1)
• Cleaner: 8% (n=2)
• Tax Consultant: 4% (n=1)
• Hotel Maid: 4% (n=1)
• Handworker: 4% (n=1)
• Information Technology Worker: 4% (n=1)
• Aircraft Technician: 4% (n=1)
• Housemaid: 4% (n=1)
• Illustrator: 4% (n=1)
• Medical Assistant: 4% (n=1)
• Industrial Machine Operator: 4% (n=1)
• Job Change Due to Pain: 4% (n=1)
